# Immunomodulatory Role of BLG-Derived Peptides Based on Simulated Gastrointestinal Digestion and DC-T Cell from Mice Allergic to Cow’s Milk

**DOI:** 10.3390/foods11101450

**Published:** 2022-05-17

**Authors:** Xin Ma, Fan Yang, Xuanyi Meng, Yong Wu, Ping Tong, Jinyan Gao, Hongbing Chen, Xin Li

**Affiliations:** 1 State Key Laboratory of Food Science and Technology, Nanchang University, Nanchang 330047, China; xinmachine@126.com (X.M.); 18720995289@163.com (F.Y.); mengxuanyilucky@163.com (X.M.); ericyo918@hotmail.com (Y.W.); tongping@ncu.edu.cn (P.T.); gaojy2013@ncu.edu.cn (J.G.); chenhongbing@ncu.edu.cn (H.C.); 2 School of Food Science & Technology, Nanchang University, Nanchang 330047, China; 3 Sino-German Joint Research Institute (Jiangxi-OAI), Nanchang University, Nanchang 330047, China; 4 Jiangxi Province Key Laboratory of Food Allergy, Nanchang University, Nanchang 330047, China

**Keywords:** cow’s milk, digested peptides, unique peptides, immunologic function

## Abstract

Peptides, but not whole protein, elicit an allergic reaction since food allergens should be consumed by digestion. In this study, we explored the remaining peptides after simulated digestion of cow’s milk in order to search for β-lactoglobulin (BLG)-derived peptides that could play an immunomodulatory role. As a major allergen in milk, BLG-derived peptides, 109 in total, were identified both from simulated infant and adult digestion in vitro. These peptides were mainly located in four regions, and they were synthesized as five peptides, namely, BLG_1–14_, BLG_24–35_, BLG_40–60_, BLG_82–101_, and BLG_123–139_. Then, the effect of peptides on the Caco-2 cell’s transport absorption, the co-stimulatory molecules of DC, and the T-cell phenotype was explored. The results suggested all peptides showed better transport absorption capacity with the apparent permeability coefficient higher than 2 × 10^−6^ cm·s^−1^. The ability of BLG_40–60_ for promoting lamina propria-derived DC cell (LPDC) maturation was observed by the increase in MHC II. Moreover, BLG_1–14_ and BLG_40–60_ directed activation of T lymphocytes towards a Th1 phenotype. This is the first report of the immunomodulatory potential of peptides in the sensitization of allergic reaction, and one peptide, BLG_40–60_, was regarded as an immunomodulatory peptide, one that should be further explored in an animal model in depth.

## 1. Introduction

Cow milk is considered a perfect source of protein and exogenous bioactive peptides, but the allergenic protein in cow’s milk limits its consumption. For infants, cow’s milk protein allergy (CMPA) is one of the most common food allergies, with an estimated prevalence in developed countries ranging from 0.5% to 3% [1], with the prevalence in China being 2.69% [2]. Usually, the casein (CN), β-lactoglobulin (BLG), and α-lactalbumin (ALA) are considered to be the main allergens in cow’s milk. In addition, human breast milk does not contain BLG, so many infants and young children are thus allergic to BLG, with 82% of people being reported to be allergic to BLG in an early work [3].

Digestion is a biochemical process by which the body absorbs nutrients, and CMPA emerges during this process. After digestion, digested polypeptides may contain allergen epitopes. It is generally believed that the longer the allergic food protein remains in the mammalian gastrointestinal tract, the more likely it will be to trigger an immune response. Picariello et al. [4] found that some intact ALA, BLG, and ^125^TPEVDDEALEK^135^ of BLG could be detected in digestive fluid after simulated gastrointestinal and intestinal brush border membrane enzyme digestion of bovine milk protein in vitro. Furthermore, digestion-resistant fragments of BLG were detected in the Caco-2 cell monolayer model and in serum derived from children with CMPA [5]. On the other hand, their studies reported that the BLG hydrolysates showed certain immune tolerance. The potential allergenicity of BLG-CN glycomacropeptide mixtures (BCGs) was detected by a human IgE binding assay after digestion, and IgE binding of the digestive products significantly reduced in the presence of BCGs [6]. Meulenbroek et al. [7] reported that oral pre-exposure to BLG-derived peptide (LLDAQSAPLRVYVEELKP) combined with a diet containing non-digestible oligosaccharides (scGOS/lcFOS/pAOS) reduced the allergic response in a mouse model for cow’s milk allergy. In follow-up studies, the potential of early oral exposure to a mixture of four synthetic BLG-derived peptides was shown to prevent allergy development in a murine model [8]. Furthermore, the tolerogenesis of BLG-derived peptides in a hydrolyzed whey-based infant formula has been demonstrated [9]. Altogether, these studies supported the hypothesis that specific amino acid regions may be suitable for specific allergic immunomodulation.

To our knowledge, the studies on the immunoreactivity of digested products mainly focused on the milk proteins, and only CN, BLG, and ALA have been studied [5,10,11]. Recently, Macierzanka et al. [12] reported the digestibility rate of BLG and β-casein increased after the pepsin digestion in emulsions, and another study reported that phosphatidylcholine could protect BLG during digestion, leading to the promotion of sensitization [13]. However, research on the whole dairy product in vitro has not been reported. Therefore, it is of great significance to study simulated digestion of commercial milk and evaluate the immunoreactivity of digestion products. In this study, we simulated the gastrointestinal digestion of milk products and explored the stability, absorption, and immunogenicity of five BLG-derived peptides, which were the major epitopes found by Benede et al. [14], Adel-Patient et al. [15], and Inoue et al. [16]. 

## 2. Materials and Methods

### 2.1. Simulated Gastric and Intestinal Digestion of Bovine Milk

Cow milk was digested according to Do et al. [17] and Minekus et al. [18]. Briefly, freeze-dried cow milk products purchased from a supermarket (the UHT for simulated adult digestion, infant formula for simulated infant digestion) was dissolved in distilled water at 30 mg of protein/mL, followed by digestion in simulated adult and infant gastrointestinal fluids in vitro. The detailed steps were as follows: the mix was diluted at a ratio of 1:1 (*v:v*) in simulated gastric fluid containing pepsin from porcine gastric mucosa (25,000 U/mL for simulated adult digestion, 3125 U/mL for simulated infant digestion [19], Sigma-Aldrich, Shanghai, China). Samples were withdrawn at 60 min during gastric digestion, and the reaction was stopped by adjusting the pH to 7 with 1 M NaHCO_3_. Half of the stomach digestion solution was mixed with the same volume of simulated intestinal fluid (total volume 100 mL) containing trypsin (5 mg/mL, Sigma-Aldrich, Shanghai, China) and porcine bile extract (0.05 M in the final mixture, Sigma-Aldrich, Shanghai, China). The intestinal phase was induced for 60 min by incubation at 37 °C in an orbital shaker at approximately 130 rpm, followed by digestion cessation using 4-(2-aminoethyl) benzenesulfonyl fluoride hydrochloride (4 mM, Sigma-Aldrich, Shanghai, China). The temperature of all sample emulsions and simulated fluids was adjusted to 37 °C before use, digestion of each sample was performed in duplicate, and the resulting products were stored at −20 °C until analysis.

### 2.2. Protein Profile after Simulated Gastric and Intestinal Digestion

After in vitro gastric and intestinal digestion, the products were detected by SDS-PAGE with 16.5% polyacrylamide gels. Gels were stained with Coomassie Blue (Sigma-Aldrich, Shanghai, China), and images were captured with a G:BOX F3 Gel Documentation System (Syngene, Cambridge, UK).

The digested products were centrifuged at 300× *g* for 10 min, and the supernatant was collected for HPLC–MS/MS. For HPLC–ESI–MS/MS analysis; the procedure was carried out in positive-ion reflectron mode. The eluents used were as follows: (A) 0.1% formic acid in water and (B) 0.1% formic acid in acetonitrile. First, samples were separated on a C18 column (75 μm i.d. × 10 cm, 3 μm, C18-A2, Thermo Scientific EASY column, Waltham, MA, USA) and analyzed at 220 nm. The injection volume was 50 μL, and the flow was set at 300 nL min^−1^. Then, MS mode acquisition was performed over the *m/z* range 300–1800, and the resolution of MS was 70,000 at 200 *m/z*. The *m/z* of the polypeptide and polypeptide fragments was determined as follows: 20 fragments were acquired after each full scan, the MS2 activation type was HCD, and the isolation window was 2 m/z. The normalized collision energy was 30 eV, and the underfill ratio was 0.1%.

### 2.3. Synthetic Peptides and Reagents

Information on BLG-derived digestive peptides is shown in Table S1. The five peptides, BLG_1–14_ (LIVTQTMKGLDIQK, A1), BLG_24–35_ (MAASDISLLDAQ, A2), BLG_40–60_ (RVYVEELKPTPEGDLEILLQK, A3), BLG_82–101_ (FKIDALNENKVLVLDTDYKK, A4), and BLG_123–139_ (VRTPEVDDEALEKFDKA, A5), were chemically synthesized by Sangon Cooperation (Shanghai, China). The peptide OVA_323–339_ (ISQAVHAAHAEINEAGR, A6) was synthesized and used as a positive control in the stimulation assay. LPS was purchased from Sigma-Aldrich (Shanghai, China).

### 2.4. Establishment of Caco-2 Monolayers and the Absorption Rate of Digested Peptides

Caco-2 cells (a human colon adenocarcinoma cell line) were preserved in our lab and cultured in DMEM supplemented with 12% (*v/v*) FBS (FND500, ExCell Bio, Shanghai, China), 1% (*v/v*) L-glutamine, 1% (*v/v*) streptomycin–penicillin, and 1% (*v/v*) nonessential amino acids at 37 °C and 5% CO_2_. Caco-2 cells were seeded in 12-well Transwell plates at a density of 2 × 10^5^ cells per well. On the 10th to 21st days, the transepithelial electrical resistance (TEER) of each well was detected using a Millicell ERS-2 V-ohm meter (Millipore, Boston, MA, USA). A cell monolayer with a TEER value higher than 400 Ω × cm^2^ was selected for further work (Figure S1A,B). After 21 days, fluorescein sodium transport permeability in normal and inflammatory modes (IL-1β was added at a final concentration of 10 ng/mL on the 21st day and removed after 2 days of culture [20]) was measured to be 1.19 μg/h·cm^2^ and 1.71 μg/h·cm^2^, respectively, which verified the integrity of the Caco-2 cell monolayer. Then, the apparent permeability coefficient (Papp) of each well was calculated at time points of 1 h, 2 h, 3 h, and 4 h. Papp values were calculated using the following equation:

Papp (cm·s^−1^) = (dQ/dt)/(A × C) (1)

where dQ/dt is the transport volume per unit time (μg/s), A is the area of the transport membrane (1.12 cm^2^), and C is the initial concentration on the AP side (μg/L).

### 2.5. Animals and Immunization Protocol

**Figure 1:**
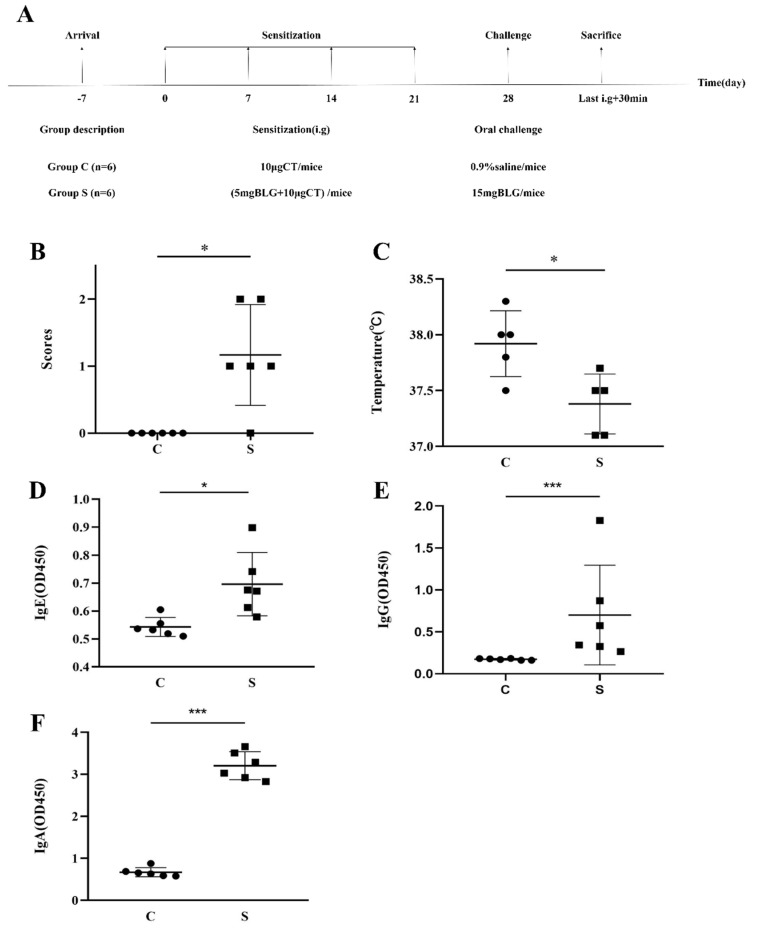
Animal experimental design and effects of eliciting dose on the allergic symptoms. (**A**) Protocols of oral administration of Balb/c mice with bovine BLG. The scores of clinical symptoms (**B**) and body temperature (**C**) were observed during 30 min after the last oral challenge. Specific antibody levels of BLG−specific IgE (**D**), IgG (**E**), and IgA (**F**) were measured by ELISA. The results are represented as the mean ± SD (*n* = 6, each point (round and square) represents data from an individual mouse; the data were calculated in three replicates; * *p* < 0.05, *** *p* < 0.01, as determined by one−way analysis of variance (ANOVA) with Duncan’s post-test).

Four-week-old female Balb/c mice (SPF) were used handled according to local animal care practices. The animal feeding certificate number was SYXK (gan) 2017-00021. Mice were randomly assigned into groups (n = 6/group). The experimental groups, protocol, and timeline are summarized in Figure 1A. On days 0, 7, 14, and 21, mice were subjected to intragastric gavage (ig) with BLG solubilized in 0.9% saline, while cholera toxin B subunit was used as the mucosal adjuvant. On day 28, the mice were sacrificed 30 min after the last challenge. Symptoms were evaluated by using a scoring system modified slightly from previous reports [21] and scored as follows: 0 = no symptoms; 1 = scratching and rubbing around the nose and head; 2 = puffiness around the eyes and mouth, pilar erecti, reduced activity, and/or decreased activity with increased respiratory rate; 3 = wheezing, labored respiration, and cyanosis around the mouth and the tail; 4 = no activity after prodding or tremor and convulsion; and 5 = death.

The allergic scores and the final temperature were detected as shown in Figure 1B and C, respectively. Sera were collected to evaluate the values of IgE, IgG, and IgA, as shown in Figure 1D–F. Small intestine tissues and MLN were removed after 30 min the last gavage under sterile conditions for further experiments. The small intestine was taken from the duodenum of the lower part of the stomach to the front of the cecum.

### 2.6. Separation of Cells in the LP

The digestive enzyme solutions used were as follows: (I) DTT (0.0308 g), EDTA (0.2924 g), and FBS (10 mL) were dissolved in 190 mL of D-HBSS and then sterilized by high-temperature and high-pressure steam for 20 min. (II) Collagenase D (0.05 g, 11088866001, Roche, Basel, Switzerland), DNaseI (10104159001, Roche, Basel, Switzerland), dispase II (0.3 g, 4942078001, Roche, Basel, Switzerland), and FBS (5%) were dissolved in 100 mL of HBSS and used under aseptic conditions. Every solution was preheated to 37 °C before use. The LP dissociation protocol was performed as follows [22,23]: after excising Peyer’s patches and residual fat tissue, the intestine was cut into pieces of approximately 0.5 cm in length and washed twice in D-HBSS. Epithelial cells were separated from the underlying LP by incubation in digestive enzyme solution I for 20 min at 37 °C with vigorous shaking. LP tissue was pulse-vortexed for 20 s and washed twice in HBSS. The remaining tissue was digested in 20 mL of digestive enzyme solution II for 20 min at 37 °C with shaking. In addition, tissue digestion was repeated twice. Leukocytes in the LP were isolated by 40% Percoll (GE Healthcare, Chicago, IL, USA) followed by centrifugation at 1100× *g* for 20 min at room temperature. The precipitate was collected for further applications.

### 2.7. Separation of Cells in Mesenteric Lymph Nodes

MLN tissue was dissected and placed on a disposable sterile culture dish and then crushed through a 70 μm cell sieve using a tissue-crushing glass rod with 10 mL of RPMI-1640 incomplete medium. All crushing solutions were collected and centrifuged at 300× *g* for 5 min at room temperature, and then the supernatant was discarded. The pellet was resuspended in RPMI-1640 incomplete medium and centrifuged, and the supernatant was discarded again, followed by the addition of 1 mL of RPMI-1640 complete medium (containing 12% FBS) for further applications.

### 2.8. CD4^+^ T Cell Sorting and CFSE Staining

MLN-derived CD4^+^ T cells were sorted by a CD4-positive sorting magnetic bead kit (anti-mouse CD4 magnetic particles-DM, 551539, BD Bioscience, Franklin Lakes, NJ, USA) according to the manufacturer’s instructions. Separation was controlled by flow cytometry (purity > 92%, Figure S2). Purified CD4^+^ T cells were prestained with CFSE (CellTrace™ CFSE Cell Proliferation Kit, C34554, Thermo Fisher, Waltham, MA, USA) before use in co-culture assays.

### 2.9. Cell Stimulation and Cytokine Measurements

For the peptide–LP cell co-culture assay, 1 × 10^5^ LP cells/mL were co-cultured in 96-well plates (NEST, Wuxi, China) in triplicate with different peptides (the final concentration was 100 ng/mL) in 100 μL of RPMI-1640 incomplete medium supplemented with 12% heat-inactivated FBS. After 72 h, the surface markers of LP cells were detected by flow cytometry.

For the peptide–MLN cell co-culture assay, 4 × 10^6^ MLN cells/mL were co-cultured in 24-well plates (NEST, Wuxi, China) in triplicate with different peptides (the final concentration was 50 μg/mL) in 1 mL of RPMI-1640 incomplete medium supplemented with 12% heat-inactivated FBS, followed by the addition of 2.5 μL of Con-A to each well. After 72 h, T cell polarization and changes in the surface markers of MLN cells were detected by flow cytometry.

For the peptide–LP cell–CD4^+^ T cell co-culture assay, 2 × 10^5^ LP cells/mL were co-cultured in 96-well plates (U-bottom, NEST, Wuxi, China) in triplicate with different peptides (the final concentration was 100 ng/mL) in 100 μL of RPMI-1640 incomplete medium supplemented with 12% heat-inactivated FBS. After 6 h, 100 μL of 1 × 10^6^ MLN-CD4^+^ T cells/mL was added to each well. After a total of 72 h of incubation, the surface markers of LP cells and T cell differentiation were detected by flow cytometry, and cytokines in the supernatant were detected by Luminex.

### 2.10. Flow Cytometry and FACS Analysis

Cells were washed with PBS containing 2% FBS, fixed, permeabilized, and stained with appropriate isotype controls and certain antibodies in the dark at 4 °C with or without a fixation/permeabilization kit (BD Cytofix/CytopermTM, Cat. 554714, BD Biosciences, Franklin Lakes, NJ, USA). Samples were analyzed using BD C6plus (BD Biosciences, Franklin Lakes, NJ, USA), and data were analyzed using FlowJo v10 software (Stanford University, Stanford, CA, USA).

### 2.11. Statistical Analysis

Statistical analysis was performed using SPSS 20.0 software (SPSS Inc., 2012, Chicago, IL, USA). Data are expressed as the means ± SDs of an experiment performed in multiple replicates. One-way ANOVA and Dunnett’s multiple comparison test were used to assess the differences between groups. Differences were considered statistically significant at *p* < 0.05.

## 3. Results

### 3.1. Protein Profile of Simulated Digestion In Vitro

**Figure 2:**
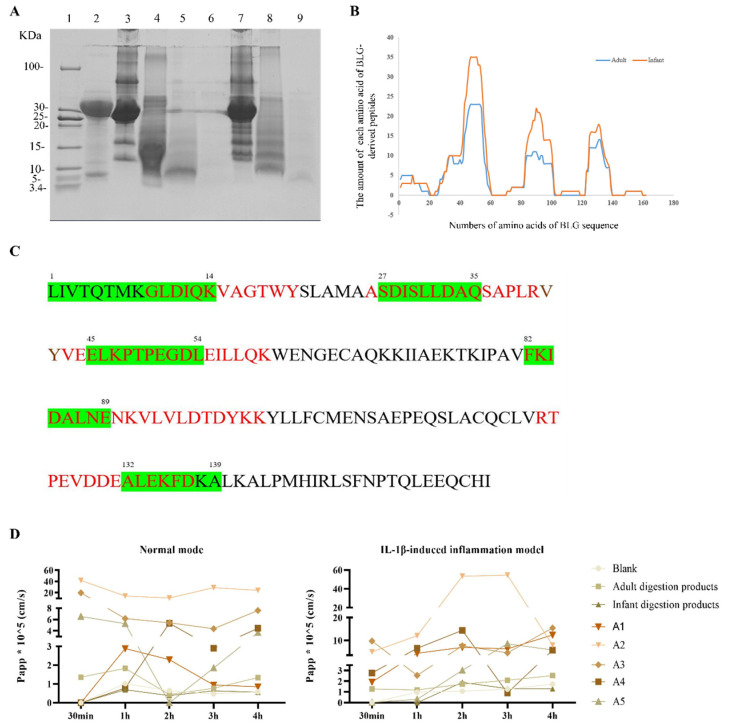
Gastric digestion of cow’s milk and the good transport ability of BLG digestion-derived peptides by the Caco-2 cell monolayer. The protein profile of cow’s milk after simulated digestion was detected by (**A**) Tricine-SDS-PAGE. Lane 1: unstained low range protein ladder. Lane 2: pepsin. Lane 3: the cow’s milk. Lane 4: cow’s milk digested in the simulated adult gastrointestinal fluid for 0 min. Lane 5: cow’s milk digested in the simulated adult gastric fluid for 60 min. Lane 6: cow’s milk digested in the simulated adult gastrointestinal fluid for 60 min. Lane 7: cow’s milk digested in the simulated infant gastrointestinal fluid for 0 min. Lane 8: cow’s milk digested in the simulated infant gastric fluid for 60 min. Lane 9: cow’s milk digested in the infant simulated gastrointestinal fluid for 60 min. (**B**) The line graph shows the distribution of BLG-derived peptide segments after simulated adult and infant digestion. Y-axis value represents the occurrence numbers of amino acids of BLG peptides. (**C**) The whole amino acid sequence of BLG used in our study; the red letters indicate the areas where unique peptides are present after the simulated digestion of the infants, while the green letters represent unique peptides in the adults’ digestion. (**D**) The Papp of BLG peptides across Caco-;2 monolayers at 30 min, 1 h, 2 h, 3 h, and 4 h in the normal model and IL-1β-induced inflammation model, respectively. The data were calculated in three replicates. BLG, beta-lactoglobulin. Papp, apparent permeability coefficients.

Tricine-SDS-PAGE (Figure 2A) showed the protein profile of the digested milk in the simulated adult and infant gastric and gastrointestinal fluids. At the same dilution ratio, the bands of gastric digestion products in infants (lanes 7 and 8) were heavier than those in adults (lanes 4 and 5), and no visible difference was identified during the gastric and gastrointestinal phases of digestion in infants (lanes 7 and 8). In addition, electrophoretic bands corresponding to BLG (MW = 18.3 kDa) and ALA (MW = 14.3 kDa) differed between the simulated adult and infant models. Thus, in the simulated infant digestion condition, the main allergen in cow milk was still present. Information on peptides with MWs below 10 kDa was collected from in vitro digests collected during the gastrointestinal phase from both adults and infant’s digestion products, followed by HPLC–MS/MS analysis under equal conditions. 

As summarized in Table 1, a total of 731 peptides belonging to milk allergen proteins were detected, approximately 109 of which (MWs between 813.42 and 2218.22 Da) corresponded to BLG. The detailed sequence information of BLG-derived digested peptides is summarized in Table S1 in the Supplementary Materials. We found that β-casein and BLG were more resistant to gastrointestinal digestion because the number of peptides from these two proteins accounted for more than 70% of all peptides, as previously described by other authors [10]. From the perspective of sequence coverage in our study, the values for β-casein and BLG were 88.4% and 72.5% in the remaining fragments, respectively. Figure 2B shows the appearance frequency of each amino acid identified as part of a peptide sequence for BLG, which provided qualitative information about the protein coverage, and the remaining peptide positions were also identified in both adult and infant models. From the line chart, the peptide profile of BLG under the infant digestion condition was similar to that in adults, which was characterized by AA1–20, AA23–60, AA70–101, and AA123–140, according to the similar abundance levels of amino acids. 

A further outcome that emerged from the data was the unique peptides remained after simulated adult digestion and infant digestion. As shown in Table 2 and Figure 2C, the peptides ^1^LIVTQTMKGLDIQ(K)^14^, ^27^SDISLLDAQ^35^, ^45^ELKPTPEGDL^54^, ^82^FKIDALNE^89^, and ^132^ALEKFDKA^139^ were found to be unique present in adult digestive conditions, compared with infant digestive conditions. Interestingly, in contrast to the unique present peptides in the infant digestive condition, two of the above peptides started from the N or C terminus of glutamic acid and two of them had the same C terminus of glutamine. However, only 20% (7 in 35) of the unique peptides with glutamine/glutamic acid as the N or C terminus were found in simulated infant digestion fluid. Combining the role of glutamine/glutamic acid for supporting immune system cells and protecting intestines, the presence of specific glutamine/glutamic acid ends in adult digestive peptides might account for the stronger gut microenvironment in adults than in infants.

Then, the peptides ^1^LIVTQTMKGLDIQK^14^ (BLG_1–14_, A1), ^24^MAASDISLLDAQ^35^ (BLG_24–35_, A2), ^40^RVYVEELKPTPEGDLEILLQK^60^ (BLG_40–60_, A3), ^82^FKIDALNENKVLVLDTDYKK^101^ (BLG_82–101_, A4), and ^123^VRTPEVDDEALEKFDKA^139^ (BLG_123–139_, A5), which were the abundant region and also contained the region of unique peptide in adult digestive condition, were synthesized for future analysis, as shown in Table 3.

### 3.2. The Absorption Rate of BLG-Derived Peptides

To evaluate the absorption abilities of digestion products and BLG synthetic peptides, a Caco-2 monolayer, a normal mode, and an IL-1β-induced inflammation model were constructed. All synthesized peptides had a better ability to penetrate the Caco-2 monolayer membrane than the simulated digestion products (Figure 2D). Within 2 h, all the Papp values of A1, A2, and A3 were higher than 2 × 10^−6^ cm·s^−1^ at the three time points, indicating that they had good absorption and transport capabilities.

### 3.3. Effects of BLG-Derived Peptides on LPDC Maturation

**Figure 3:**
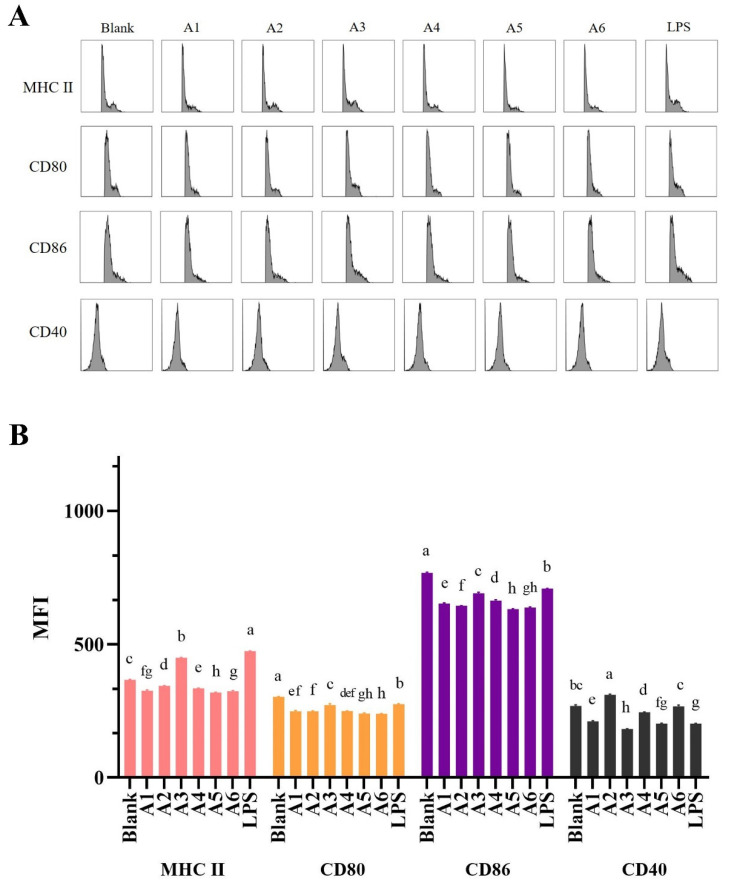
BLG_40–60_ promoted the maturation of LP cells in the peptide–LP cell assay. (**A**) The histogram results of MHC II, CD80, CD86, and CD40 of LP cells with different stimulation of peptides or LPS. (**B**) Bar graphs showing the MFI levels of MHC II, CD80, CD86, and CD40 summarized from (**A**), respectively. The results are represented as the mean ± SD (*n* = 3, the data were calculated in three replicates, the same letters indicate the lack of significance of the MHC II/CD80/CD86/CD40 column, as determined by two-way analysis of variance (ANOVA) with Duncan’s post-test (*p* < 0.05)). LP cells, intestinal lamina propria cells. MHC II, MHC classic molecule II. MFI, median fluorescence intensity.

**Figure 4:**
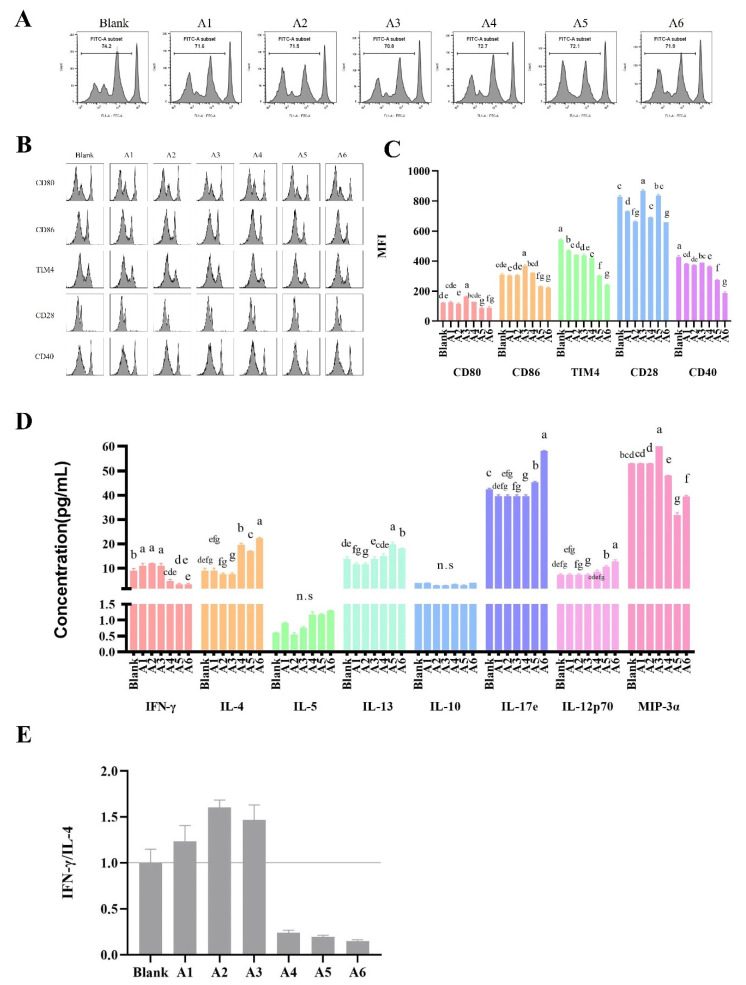
Peptide BLG_40–60_ showing the promote maturation ability of LP cells and directed differentiation of activated CD4^+^ T lymphocytes towards a Th1 phenotype, but not the proliferation, in the peptide–LP cell–MLN cell-co-culture assay. (**A**) The intensity of CFSE staining after stimulation. (**B**) The histogram results of CD80, CD86, TIM-4, CD28, and CD40. Each row corresponds to a different gate result, and each column corresponds to a different peptide treatment group. (**C**) Bar graphs showing the MFI levels summarized from (**B**). (**D**) Bar graphs showing the cytokines levels of IFN-γ, IL-4, IL-5, IL-13, IL-10, IL-17e, IL-12p70, and MIP-3α. (**E**) Bar graphs showing the IFN-γ/IL-4 ratio summarized from (**D**). The results are represented as the mean ± SD. (The data were calculated in three replicates for flow cytometry assay, and two replicates for cytokine level. The same letters indicate the lack of significance, as determined by two-way analysis of variance (ANOVA) with Duncan’s post-test (*p* < 0.05).) LP cell, intestinal lamina propria cell. Th, T helper lymphocyte. MLN, mesenteric lymph node. CFSE, 5-(and 6)-carboxyfluorescein diacetate, succinimidyl ester. TIM-4, T cell immunoglobulin and mucin domain (TIM)-4. MFI, median fluorescence intensity. IFN-γ, interferon γ. IL, interleukin.

To obtain BLG-sensitized cells, we constructed BLG-allergic mice as described above (Figure 1). In the peptide-LP cell co-culture assay, the expression trends of MHC II, CD80, and CD86 in LPDCs showed a decrease after being stimulated with peptides, except the level of MHC II when treated with A3 (Figure 3B), indicating the promoting effect of A3 for LPDC cell maturation. It has been demonstrated that CD40 engagement on the surface of DCs promotes their cytokine production and the induction of costimulatory molecules on their surface, as well as facilitating the cross-presentation of antigens [24]. From the MFI of CD40-LP cells, the only increased value was detected at the A2 stimulation group, indicating A2 was not conducive to stimulating LPDC maturation but facilitated antigen delivery. However, the MFI of CD40 was decreased in the peptide–LP cell–MLN-CD4^+^ T cell co-culture assay (Figure 4B,C) when stimulated with A2, indicating the required role of T cells.

**Figure 5:**
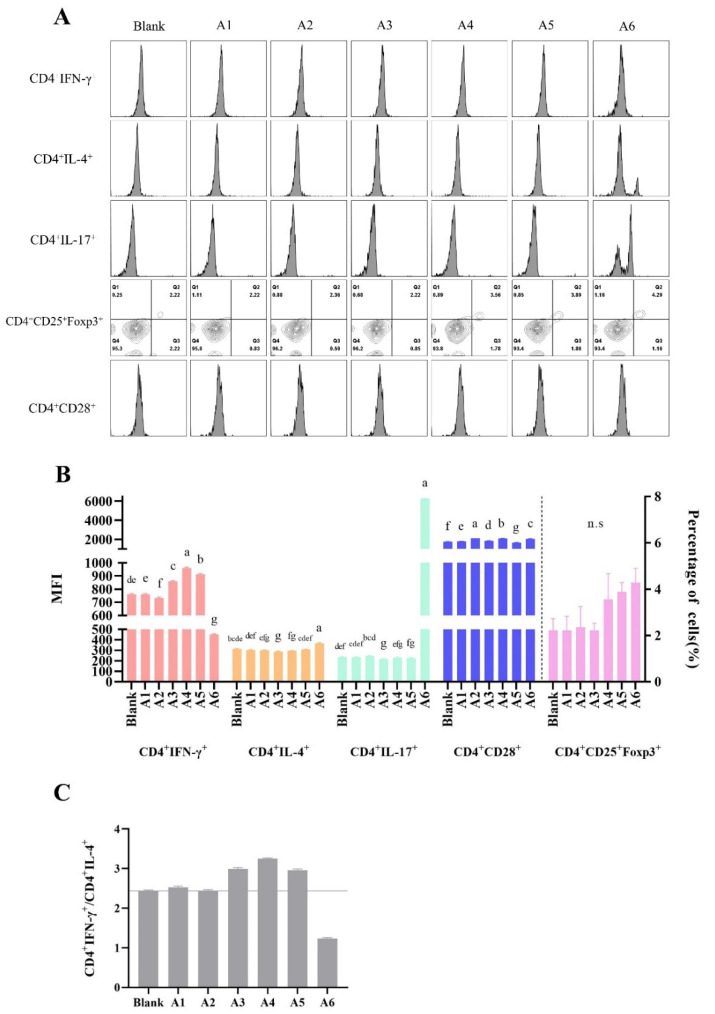
Peptides BLG_1–14_, BLG_40–60_, BLG_82–101_, and BLG_123–139_ directed differentiation of activated MLN-CD4^+^ T lymphocytes towards a Th1 phenotype in the peptide–MLN cell-co-culture assay. (**A**) The histogram results of CD4^+^IFN-γ^+^, CD4^+^IL-4^+^, CD4^+^IL-17e^+^, and CD4^+^CD28^+^, and the contour plot of CD4^+^CD25^+^Foxp3^+^ after peptide stimulation. Each row corresponds to a different gate result, and each column corresponds to a different peptide treatment group. (**B**) Bar graphs showing the MFI levels summarized from (**A**). (**C**) Bar graphs showing the CD4^+^IFN-γ^+^/CD4^+^IL-4^+^ ratio summarized from (**B**). The results are represented as the mean ± SD (*n* = 3, the data were calculated in three replicates, the same letters indicate the lack of significance, as determined by two-way analysis of variance (ANOVA) with Duncan’s post-test (*p* < 0.05)). MLN, mesenteric lymph node. Th, T helper lymphocyte. MFI, median fluorescence intensity.

Furthermore, the co-culture assay of peptide–LP cell–MLN-CD4^+^ T cells is shown in Figure 4. The ability to promote DC maturation after stimulation with A1, A2, and A4 was similar by the expression of CD80 and CD86, while A3 increased the expression of CD80 and CD86. Interestingly, in the co-culture assays of peptide–LP cells and peptide–LP cell–MLN-CD4^+^ T cells, A3 increased CD80/CD86 expression, indicating that the peptide A3 stimulated the pathway selectivity of DC cells. MIP-3α is responsible for inducing the migration of immature dendritic cells, effector, or memory T cells, and B cells [25]. From Figure 5D, the significant increased secreted cytokine of MIP-3α was detected after A3 treatment; this was another cue for the promotion role of A3 for biological functions of DC, while the A4 and A5 treatment showed the opposite role compared with the A3 group.

Taken together, an increasing trend for LPDC maturation was detected with the application of A3 in mice with sensitization in the peptide–LP cell co-culture assay.

### 3.4. The Effects of BLG-Derived Peptides on the Proliferation and Polarization of CD4^+^ T Cells

The proliferation of MLN-CD4^+^ T cells was characterized using CFSE staining (Table 4) and flow cytometry analysis (Figure 4A) after peptide stimulation. Interestingly, all peptides did not promote the proliferation of MLN-CD4^+^ T cells, while the value of CFSE of A3 was the lowest.

Furthermore, synthetic peptides had different effects on the ability to promote the polarization of MLN-CD4^+^ T cells (Figure 5). The reduction in the IFN-γ/IL-4 ratio was found upon stimulation with A2 and A6, while an increase occurred with A1, A3, A4, and A5 stimulation, suggesting a potential allergic role of A2 and A6. Peptides A1, A3, A4, and A5 showed an ability to direct the differentiation of activated T lymphocytes toward a Th1 phenotype. In addition, the trend change of CD4^+^IL-4^+^ and CD4^+^IL-17e^+^ phenotype was similar upon stimulation with peptides, indicating that the response to BLG sensitization was related to Th17. As unexpected, no significant change in CD4^+^CD25^+^Foxp3^+^ cells were seen in Figure 5B.

Next, changes in the peptide–LP cell–MLN-CD4^+^ T cell co-culture assay results for the cytokines of T cells after peptide stimulation were detected, as shown in Figure 4D and E. The increase in the IFN-γ/IL-4 ratio was found with A1, A2, and A3 treatment, which directly promoted the differentiation of activated T lymphocytes toward a Th1 phenotype. The IFN-γ/IL-4 ratio decreased with treatment with A4 or A5, opposite to the result of the single MLN-CD4^+^ T cell assay (Figure 5C), and the increased values of IL-13 and IL-17e were identified with A4 and A5 stimulation, indicating the potential allergic role of these two peptides, compared with other peptide group. In addition, higher expression levels of CD28 after stimulation with A3 and A5 were identified, indicating that A3 and A5 had a strong ability to stimulate T cells, but the two peptides cause an opposite direction of T-cell differentiation.

Taken together, in this study, the peptides A1 and A3 promoted the differentiation of activated T lymphocytes toward a Th1 phenotype, while A2, A4, and A5 showed different roles in the polarization of CD4^+^ T cells after co-culture assay in vitro, which were related to the microenvironment of intestinal immunity.

## 4. Discussion

Most digested food proteins will be degraded to form peptides or amino acids that are easily absorbed by the small intestine, which may also contain allergic epitopes. In our study, digested peptides were analyzed by HPLC–MS/MS after establishing the Caco-2 cell monolayer model. Furthermore, BLG was one of the major milk allergens and it was resistant to gastric acid, as shown above and in previous works [10]. The peptide profiles obtained from simulations of adult and infant gastrointestinal digestion in vitro were mainly concentrated in the regions BLG_1–14_, BLG_30–40_, BLG_41–60_, BLG_81–103_, and BLG_123–140_. Among them, BLG_41–60_ had the highest abundancy. In fact, previous studies have also shown that BLG_41–60_ was detected frequently in both human jejunal contents [10,14] and piglet jejunum contents [26]. BLG_41–60_ and BLG_42–56_ were identified as major T-cell epitopes in early years [27], and Adel-Patient et al. [15] further demonstrated the immunomodulatory potential of BLG_41–60_. Moreover, BLG_41–60_ and BLG_127–144_, similarly to the results of Martinez et al. [6], showed a higher abundance, as analyzed by RP–HPLC–ESI–MS/MS, in the digested mixtures. Even though these regions showed a high amount, a lower IgE-binding ability was observed, indicating lower sensitization of the areas BLG_41–60_ and BLG_127–144_. In addition, peptides BLG_43–68_ and BLG_122–146_ reported in earlier research were regarded as immune-reactive areas [14], which were also detected in our work. Taken together, the digested peptides in our experiment were similar to the reported peptide profile after simulated digestion, as shown above, indicating that the residues after digestion had potential allergic sensitization. 

Furthermore, we found that the BLG-derived peptides ^1^LIVTQTMKGLDIQ(K)^14^, ^27^SDISLLDAQ^35^, ^45^ELKPTPEGDL^54^, ^82^FKIDALNE^89^, and ^132^ALEKFDKA^139^ were found to be uniquely present in the adult digestive condition, and a total 35 of unique peptides were found in the infant digestive condition, as shown in Table 2. In detail, the region merged by unique peptides remaining in infant digestion covered the five unique peptides found in adult digestion. This was consistent with the results of protein coverage in both adult and infant models. The most interesting aspect of the data was the characteristics of the N or C terminus of the unique peptides present in adult digestion—two of the five peptides started from the N or C terminus of glutamine acid (Glu) and two of them had the same C terminus of glutamine (Gln). Glu and Gln could be converted in the body. As a predominant amino acid in the body, Gln contributed more than 50% of the total intracellular free α-amino acid pool in the skeletal muscle and blood [28]. Studies showed that Gln played a key role in the intestinal health by serving as a crucial metabolic fuel for all fast-dividing cells, as well as acting as a precursor of glutathione, pyrimidines, and purines [29,30,31]. In addition, some studies on Gln/Glu supplement diets supported the views of the benefits of Gln/Glu in intestinal health [32,33,34,35]. Thus, Gln promotes the synthesis of protein and inhibits protein catabolism in enterocytes [32]. In our study, a preference for N-terminal or C-terminal being Gln/Glu could be used as evidence of a stronger gastrointestinal tract in adults when compared with infants. Then, the peptides A1–A5, which covered the high-frequency digestion peptides and contained the regions of unique peptides in adult digestive condition, were synthesized for the analysis of immunologic effect. Few studies were available on the effect of peptides on the maturity of LP cells as well as the proliferation and differentiation of T cells, and most studies involved intact protein. MLN is the “first pass” organ for nutrients and microbial substances entering the lymph fluid in the LP. When CD103+ DCs traffic allergens from the intestine to the MLN, they could lead to the initiation of adaptive immune responses. That is the migration of DCs and cytokines existing in the intestinal microenvironment contribute to promote the proliferation and differentiation of naive T cells in MLN. Thus, MLN-T cells play a crucial role in the immune responses to cow’s milk, including those of both allergic and protective types. It is worth mentioning that epigenetics also plays a crucial role there [36,37]. Therefore, a BLG sensitization mouse model was performed for obtaining LP cells and MLN-derived CD4^+^ T cells.

Furthermore, the different experimental groups of allergen dosage were speculated to result in different mixtures of the original immune cells. The immune microenvironment in intestinal mucosal tissues plays a vital role in T-cell differentiation polarization. As reported by Kroghsbo et al. [38] and Adel-Patient et al. [39], dose-dependent effects on the specific antibody response were clearly evident in Balb/c mouse trials, and the kinetics of the response were heavily affected by the antigen-eliciting dose. Animal models may enable more intuitive and comprehensive evaluations of allergenicity; therefore, the distribution of focal immune cells requires more in-depth research under various eliciting doses or other conditions.

## 5. Conclusions

In conclusion, the peptide profile of BLG was similar to simulated gastrointestinal tract digestion in vitro both for infants and adults, and the remaining peptides were located in AA1–20, AA23–60, AA70–101, and AA123–140. Five peptides were synthesized and investigated by cytological evaluations of Caco-2 cell transport absorption, DC maturation, and T-cell proliferation and differentiation. We identified one peptide, BLG_40–60_, that promoted LPDC maturation and directed the proliferation of activated T lymphocytes toward a Th1 phenotype. The peptides also need to confirm their role further, which can be explored with their effect on other immune cells and animal models.

## Figures and Tables

**Table 1:** The protein profile (score ≥ 20) of digested products characterized by liquid chromatography–mass spectrometry. CN, casein. BLG, β-lactoglobulin. ALA, α-lactalebumin.

Protein	Unique Peptides	Sequence Coverage (%)	Mass Range (Da)	Score Range (>20)
BLG	109	72.5	813.42–2218.22	20.12–172.74
ALA	23	43.7	843.41–1596.69	22.01–106.91
α_s1_-CN	210	79.4	842.45–2687.35	20.14–245.55
α_s2_-CN	79	61.7	811.44–1768.95	20.97–203.70
β-CN	217	88.4	837.44–2845.34	20.03–176.25
κ-CN	58	62.6	776.39–2138.14	20.70–106.42

**Table 2:** The sequences of unique peptides of BLG remaining in infant digestive condition and adult digestive condition, respectively. The red letters in the infant column indicated that fragments of unique peptides existed in the simulated adult digestive condition that could be found intact in the infant digestive peptide segment. BLG, β-lactoglobulin.

Unique Peptides in Infant Digestive Condition	Unique Peptides in Adult Digestive Condition
TPEVDDEALEKF	SDISLLDAQSAPLRV
TPEVDDEALEKFD	KPTPEGDL
VEELKPTPEGD	ALEKFDKA
KIDALNENKVLVLDTDYKK	SDISLLDAQ
PEVDDEALEKFDK	FKIDALNE
VYVEELKPTPEGDLE	ELKPTPEGDL
LNENKVLV	LIVTQTMKGLDIQK
VYVEELKPTPEGDL	LIVTQTMKGLDIQ
VEELKPTPEGDLEILLQK	
VYVEELKPTPEGDLEIL	
KPTPEGDLE	
FKIDALNENKV	
NKVLVLDTDYK	
VLVLDTDYKKY	
IDALNENKVLVLDTDYK	
ELKPTPEGDLEI	
DALNENKV	
KPTPEGDLEIL	
GLDIQKVAGT	
NENKVLVLDTDY	
YVEELKPTPE	
ASDISLLDAQSAPL	
VEELKPTPE	
FKIDALNENKVL	
SLLDAQSAPLR	
GLDIQKVAGTWY	
LKPTPEGDLEI	
MAASDISLLDAQSAPL	
RVYVEELKPTPEGD	
ALNENKVL	
YVEELKPTPEGDLEILLQ	
LKPTPEGDLEILLQ	
MENSAEPEQSLA	
RTPEVDDEA	
DALNENKVLV	

**Table 3:** The information of synthetic peptides.

Peptides	Amino Acid Sequences	Purity
A1(BLG-AA_1–14_)	LIVTQTMKGLDIQK	>90%
A2(BLG-AA_24–35_)	MAASDISLLDAQ	>90%
A3( BLG-AA_40–60_)	RVYVEELKPTPEGDLEILLQK	>90%
A4(BLG-AA_82–101_)	FKIDALNENKVLVLDTDYKK	>90%
A5(BLG-AA_123–139_)	VRTPEVDDEALEKFDKA	>90%
A6(OVA-AA_323–339_)	ISQAVHAAHAEINEAGR	>90%

**Table 4:** Proliferation of CFSE-positive MLN cells (72 h proliferation assay) was evaluated by flow cytometry. The results were represented as the mean ± SD (*n* = 3, whereas different letters indicate significance in each column, as determined by two-way analysis of variance (ANOVA) with Duncan’s post-test (*p* < 0.05). SD, standard deviation). CFSE, 5-(and 6)-carboxyfluorescein diacetate, succinimidyl ester. MLN, mesenteric lymph node.

Group	Percentage of MLN-T
Blank	74.35 ± 0.21 ^a^
A1	71.80 ± 0.28 ^e,f^
A2	71.65 ± 0.21 ^f^
A3	70.90 ± 0.14 ^g^
A4	72.80 ± 0.14 ^b^
A5	72.1 ± 0.00 ^c,d,e^
A6	71.95 ± 0.07 ^d,e,f^

## Data Availability

The data presented in this study are available on request from the corresponding author.

## Supplementary Materials

The following supporting information can be downloaded at: https://www.mdpi.com/article/10.3390/foods11101450/s1, Table S1: the amino acid sequence information of BLG-derived digested products with a molecular weight below 10kDa by Liquid phase-mass spectrometry. pH1 signal column, the electrical signals of products simulated digested by adults. pH3 signal column, the electrical signals of products simulated digested by infants. BLG, beta-lactoglobulin.; Figure S1: The TEER value of the Caco-2 monolayers at each wells as described in (A) the normal model without the IL-1β-induced inflammation model. (B) The inflammation model with the application of IL-1β (final concentration was 10 ng/mL). TEER, the trans-epithelial electrical resistance.; Figure S2 The percentages of MLN- CD4+ T cells after magnetic sorting: positive cells (A) and negative cells (B).

## Abbreviations

BLG	β-Lactoglobulin
LP	Lamina propria cells
CMPA	Cow’s milk protein allergy
ALA	α-Lactalbumin
BCGs	BLG–casein glycomacropeptide mixtures
MLN	Mesenteric lymph nodes
DC(s)	Dendritic cell(s)
LPDCs	LP-derived DC cells
OVA	Ovalbumin
DMEM	Dulbecco’s modified Eagle’s medium
FBS	Fetal bovine serum
TEER	Transepithelial electrical resistance
Papp	Apparent permeability coefficient
Ig	Immunoglobulin
DTT	Dithiothreitol
EDTA	Ethylene diamine tetraacetie acid
D-HBSS	Hank’s balanced salt solution without calcium and magnesium
RPMI-1640	Roswell Park Memorial Institute 1640
Con-A	Concanavalin A
MWs	Molecular weights
CN	Casein
