# Supplementary material for: Immunomodulatory Role of BLG-Derived Peptides Based on Simulated Gastrointestinal Digestion and DC-T Cell from Mice Allergic to Cow’s Milk
Source: Foods. 2022 May 17;11(10):1450. doi: 10.3390/foods11101450 (PMC9140701; doi:10.3390/foods11101450)

Table S1. The amino acid sequence information of BLG-derived digested products with a molecular weight below 10kDa by liquid phase mass spectrometry. pH1 signal column, the electrical signals of products simulated digested by adults. pH3 signal column, the electrical signals of products simulated digested by infants. BLG, beta-lactoglobulin.

Protein	Sequence	Mass	Score	pH1 signal	pH3 signal
BLG	TPEVDDEALEKF	1391.65	172.74	0.00E+00	7.08E+06
BLG	VEELKPTPEGDLE	1454.71	148.62	1.49E+08	2.98E+08
BLG	YVEELKPTPEGD	1375.65	140.24	4.99E+07	5.34E+07
BLG	TPEVDDEALEKFDK	1634.77	133.48	4.50E+08	1.08E+09
BLG	TPEVDDEALEKFD	1506.67	132.97	0.00E+00	2.78E+06
BLG	YVEELKPTPEGDL	1488.73	131.69	1.35E+09	7.27E+08
BLG	TPEVDDEALEKFDKA	1705.80	127.18	6.53E+06	3.53E+07
BLG	VEELKPTPEGD	1212.59	123.67	0.00E+00	4.42E+07
BLG	SDISLLDAQSAPLRV	1583.85	118.52	4.64E+06	0.00E+00
BLG	IDALNENK	915.47	111.17	2.94E+07	3.46E+07
BLG	LDAQSAPLR	969.52	105.52	4.46E+06	8.00E+06
BLG	VLDTDYKK	980.52	105.14	1.37E+07	5.50E+07
BLG	KIDALNENKVL	1255.71	105.13	1.92E+08	2.86E+08
BLG	VEELKPTPEGDL	1325.67	104.24	8.02E+08	8.02E+08
BLG	EELKPTPEGDLE	1355.65	101.39	4.82E+06	1.38E+07
BLG	KIDALNENKVLVLDTDYKK	2218.22	101.38	0.00E+00	3.94E+06
BLG	PEVDDEALEKFDK	1533.72	99.84	0.00E+00	4.83E+06
BLG	VRTPEVDDEALEKFDK	1889.94	99.75	2.43E+07	3.82E+07
BLG	EALEKFDK	978.50	99.14	2.11E+08	5.58E+07
BLG	ELKPTPEGDLEIL	1452.77	98.41	2.59E+06	3.38E+07
BLG	YVEELKPTPEGDLE	1617.78	98.34	3.90E+08	4.14E+08
BLG	FKIDALNENK	1190.63	97.73	7.65E+06	3.41E+07
BLG	IVTQTMKG	876.47	96.49	2.11E+07	3.18E+08
BLG	VEELKPTPEGDLEILLQ	1922.02	92.38	2.95E+06	7.06E+06
BLG	LIVTQTMK	932.54	91.32	1.79E+08	9.80E+07
BLG	SLLDAQSAPLRV	1268.71	90.69	7.09E+06	1.19E+07
BLG	DEALEKFDK	1093.53	90.15	2.44E+07	2.21E+07
BLG	VYVEELKPTPEGDLE	1716.85	89.51	0.00E+00	9.98E+06
BLG	KIDALNENKVLVLDTDYK	2090.13	89.34	2.06E+06	9.41E+06
BLG	TPEVDDEALEK	1244.58	89.30	3.31E+07	8.29E+05
BLG	EKTKIPAV	884.53	89.30	6.03E+07	8.72E+07
BLG	ELKPTPEGDLE	1226.60	89.17	1.40E+08	1.05E+08
BLG	LNENKVLV	927.54	88.37	0.00E+00	1.48E+07
BLG	KIDALNENKVLV	1354.78	87.83	2.51E+08	6.88E+07
BLG	LVLDTDYKK	1093.60	87.48	1.19E+07	3.57E+07
BLG	DAQSAPLRV	955.51	85.68	3.71E+07	1.65E+08
BLG	VYVEELKPTPEGDL	1587.80	85.54	0.00E+00	1.57E+07
BLG	NENKVLVLDTDYKK	1677.89	85.16	3.20E+06	7.26E+06
BLG	TPEVDDEAL	987.44	84.57	6.62E+07	1.18E+08

BLG	VRTPEVDD	929.45	84.50	1.57E+08	2.86E+07
BLG	IIAEKTKIPAV	1181.74	82.26	4.48E+06	7.60E+06
BLG	YVEELKPTPEGDLEI	1730.86	80.75	1.18E+06	1.01E+07
BLG	VEELKPTPEGDLEILLQK	2050.12	80.46	0.00E+00	1.49E+07
BLG	EELKPTPEGDL	1226.60	79.66	3.17E+07	3.83E+07
BLG	VEELKPTPEGDLEI	1567.80	77.78	3.25E+06	1.27E+07
BLG	VYVEELKPTPEGDLEIL	1943.01	77.75	0.00E+00	1.86E+07
BLG	TPEVDDEA	874.36	76.85	3.79E+07	5.57E+07
BLG	EALEKFDKA	1049.54	76.28	5.82E+06	7.08E+06
BLG	KPTPEGDL	855.43	76.06	5.10E+07	0.00E+00
BLG	KPTPEGDLE	984.48	74.46	0.00E+00	2.90E+07
BLG	RVYVEELKPTPEGDL	1743.90	72.62	1.41E+07	1.12E+07
BLG	VRTPEVDDEA	1129.53	72.32	1.01E+08	4.22E+07
BLG	FKIDALNENKV	1289.70	72.29	0.00E+00	9.61E+06
BLG	LVLDTDYK	965.51	71.69	1.51E+07	3.23E+07
BLG	VLVLDTDYKK	1192.67	68.63	9.55E+07	1.77E+06
BLG	VRTPEVDDEAL	1242.61	68.54	1.45E+08	7.17E+07
BLG	NKVLVLDTDYK	1306.71	68.36	0.00E+00	4.71E+06
BLG	LSFNPTQLEEQ	1304.62	67.39	5.02E+06	7.69E+06
BLG	LDAQSAPLRV	1068.59	67.33	1.14E+08	2.11E+08
BLG	KIDALNEN	915.47	66.50	1.49E+07	1.56E+07
BLG	LIVTQTMKG	989.56	63.22	4.36E+07	9.32E+06
BLG	VLVLDTDYKKY	1355.73	63.07	0.00E+00	3.52E+06
BLG	ALEKFDKA	920.50	62.58	8.94E+06	0.00E+00
BLG	VRTPEVDDE	1058.49	61.78	1.59E+08	3.44E+07
BLG	KIDALNENK	1043.56	61.56	3.55E+08	3.51E+08
BLG	VRTPEVDDEALE	1371.65	61.11	4.18E+06	2.86E+06
BLG	SDISLLDAQ	960.48	60.89	1.82E+07	0.00E+00
BLG	IDALNENKVLVLDTDYK	1962.03	60.49	0.00E+00	1.59E+06
BLG	ELKPTPEGDLEI	1339.69	60.26	0.00E+00	4.58E+06
BLG	TPEVDDEALE	1116.48	58.25	1.29E+06	7.83E+06
BLG	FKIDALNE	948.49	56.92	2.20E+06	0.00E+00
BLG	LKPTPEGDL	968.52	56.26	3.79E+08	4.01E+08
BLG	IDALNENKVLV	1226.69	55.31	1.29E+07	9.15E+06
BLG	NKVLVLDTDYKK	1434.81	55.26	1.75E+06	1.03E+07
BLG	KIDALNENKV	1142.63	54.78	2.03E+08	8.06E+08
BLG	YVEELKPTPEGDLEIL	1843.95	53.30	8.86E+06	3.94E+07
BLG	ELKPTPEGDL	1097.56	52.58	5.75E+08	0.00E+00
BLG	VLVLDTDYK	1064.58	52.58	5.87E+07	1.13E+08
BLG	DALNENKV	901.45	51.86	0.00E+00	3.87E+06
BLG	VEELKPTPEGDLEIL	1680.88	51.77	3.18E+07	1.28E+08
BLG	YVEELKPTPEGDLEILLQK	2213.18	47.68	1.02E+06	2.25E+06
BLG	KPTPEGDLEIL	1210.64	45.83	0.00E+00	2.06E+07
BLG	LDAQSAPL	813.42	45.68	3.54E+08	2.14E+08

BLG	RVYVEELKPTPEGDLE	1872.95	45.54	3.54E+06	9.96E+06
BLG	GLDIQKVAGT	1000.56	45.16	0.00E+00	7.87E+06
BLG	RVYVEELKPTPEGDLEIL	2099.12	45.00	1.11E+06	3.52E+06
BLG	NENKVLVLDTDY	1421.70	44.61	0.00E+00	9.07E+05
BLG	SDISLLDAQSAPL	1328.68	43.68	9.38E+07	1.06E+08
BLG	YVEELKPTPE	1203.60	43.31	0.00E+00	1.30E+06
BLG	GLDIQKVAGTW	1186.63	39.79	4.21E+06	5.33E+06
BLG	ASDISLLDAQSAPL	1399.72	39.73	0.00E+00	9.45E+06
BLG	VEELKPTPE	1040.54	36.59	0.00E+00	1.59E+06
BLG	FKIDALNENKVL	1402.78	35.66	0.00E+00	2.01E+06
BLG	SLLDAQSAPLR	1169.64	34.96	0.00E+00	1.60E+06
BLG	ISLLDAQSAPLRV	1381.79	32.93	2.05E+05	2.77E+05
BLG	LIVTQTMKGLDIQK	1586.91	31.68	2.20E+06	0.00E+00
BLG	GLDIQKVAGTWY	1349.70	31.35	0.00E+00	1.21E+06
BLG	LIVTQTMKGLDIQ	1458.81	31.22	8.74E+05	0.00E+00
BLG	LKPTPEGDLEI	1210.64	30.89	0.00E+00	3.90E+06
BLG	LKPTPEGDLE	1097.56	30.61	1.12E+08	1.65E+08
BLG	MAASDISLLDAQSAPL	1601.80	28.81	0.00E+00	1.85E+06
BLG	RVYVEELKPTPEGD	1630.82	28.28	0.00E+00	2.16E+06
BLG	ALNENKVL	899.51	27.74	0.00E+00	1.27E+06
BLG	VEELKPTPEGDLEILL	1793.97	26.77	1.18E+06	3.73E+06
BLG	YVEELKPTPEGDLEILLQ	2085.09	24.48	0.00E+00	6.99E+05
BLG	LKPTPEGDLEILLQ	1564.87	24.43	0.00E+00	6.35E+05
BLG	MENSAEPEQSLA	1304.56	20.83	0.00E+00	2.08E+06
BLG	RTPEVDDEA	1030.46	20.78	0.00E+00	1.19E+06
BLG	DALNENKVLV	1113.60	20.21	0.00E+00	2.93E+06

Figure S1. The TEER value of the Caco-2 monolayers at each wells, as described in (A) the normal model without the IL-1β-induced inflammation model. (B) The inflammation model with the application of IL-1β (final concentration was 10 ng/mL). TEER, trans-epithelial electrical resistance.

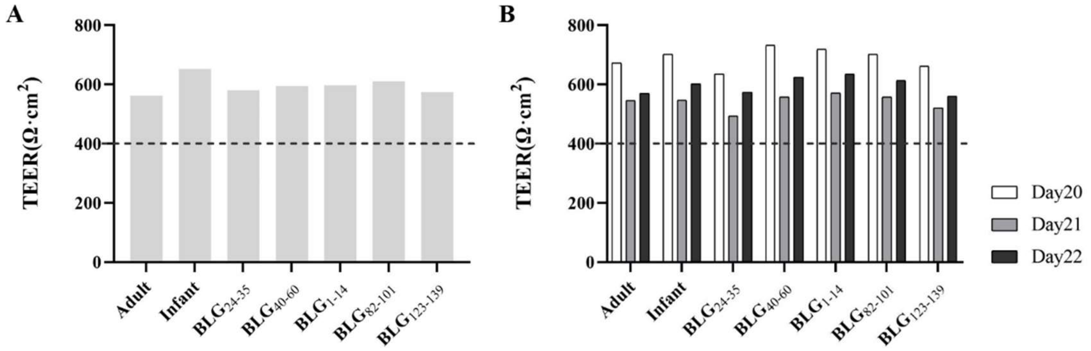

Figure S2 The percentages of MLN- $CD4^+$ T cells after magnetic sorting: positive cells (A) and negative cells (B).

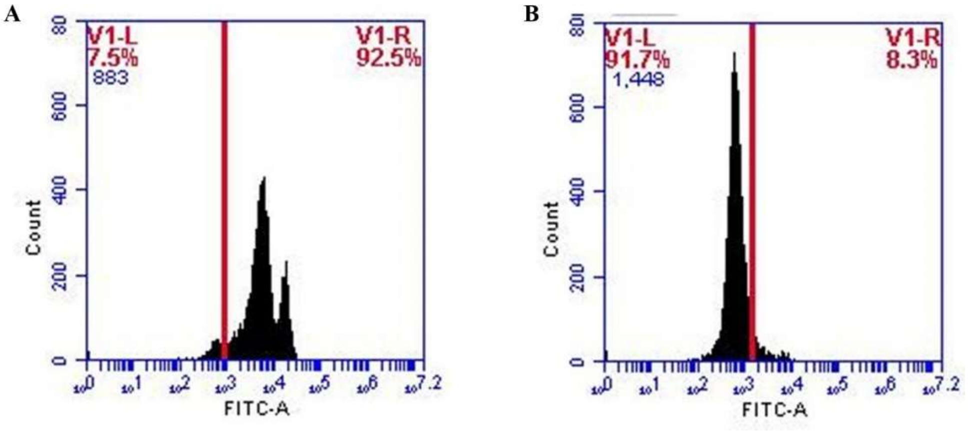

Supplement: Supplementary file 1 [file foods-11-01450-s001.zip › foods-1681784-supplementary.pdf]
